# Stable Isotope and Metagenomic Profiling of a Methanogenic Naphthalene-Degrading Enrichment Culture

**DOI:** 10.3390/microorganisms6030065

**Published:** 2018-07-10

**Authors:** Courtney R. A. Toth, Carolina Berdugo-Clavijo, Corynne M. O’Farrell, Gareth M. Jones, Andriy Sheremet, Peter F. Dunfield, Lisa M. Gieg

**Affiliations:** Department of Biological Sciences, University of Calgary, 2500 University Drive NW, Calgary, AB T2N 1N4, Canada; courtney.toth@utoronto.ca (C.R.A.T.); cberdugo@ucalgary.ca (C.B.-C); cmofarre@ucalgary.ca (C.M.O.); gmjones@ucalgary.ca (G.M.J.); andriy.sheremet@ucalgary.ca (A.S.); pfdunfie@ucalgary.ca (P.F.D.)

**Keywords:** naphthalene, methanogenesis, polycyclic aromatic hydrocarbons, anaerobic degradation, bioremediation, stable isotope probing, metagenomics

## Abstract

Polycyclic aromatic hydrocarbons (PAH) such as naphthalene are widespread, recalcitrant pollutants in anoxic and methanogenic environments. A mechanism catalyzing PAH activation under methanogenic conditions has yet to be discovered, and the microbial communities coordinating their metabolism are largely unknown. This is primarily due to the difficulty of cultivating PAH degraders, requiring lengthy incubations to yield sufficient biomass for biochemical analysis. Here, we sought to characterize a new methanogenic naphthalene-degrading enrichment culture using DNA-based stable isotope probing (SIP) and metagenomic analyses. 16S rRNA gene sequencing of fractionated DNA pinpointed an unclassified *Clostridiaceae* species as a putative naphthalene degrader after two months of SIP incubation. This finding was supported by metabolite and metagenomic evidence of genes predicted to encode for enzymes facilitating naphthalene carboxylic acid CoA-thioesterification and degradation of an unknown arylcarboxyl-CoA structure. Our findings also suggest a possible but unknown role for *Desulfuromonadales* in naphthalene degradation. This is the first reported functional evidence of PAH biodegradation by a methanogenic consortium, and we envision that this approach could be used to assess carbon flow through other slow growing enrichment cultures and environmental samples.

## 1. Introduction

Polycyclic aromatic hydrocarbons (PAH) are ubiquitous environmental pollutants generated during the incomplete combustion of organic materials (e.g., fossil fuels, refined fuel products, and wood), but also from natural seepage of petroleum or coal deposits, accidental fuel releases, and volcanic activities [1]. Due to their high hydrophobicity, PAHs tend to partition and deposit in sedimentary environments, but water-soluble components (e.g., 2-to 3-ringed PAHs) are also capable of migrating through anoxic groundwater systems [2]. Given that PAHs exhibit toxigenic, mutagenic, and/or carcinogenic properties [1,3], PAH-contaminated aquifers pose a significant threat to ecological and human health. This issue has prompted more than 40 years of field- and microcosm-based research documenting the extent of PAH mineralization. As a result, it is firmly established that two- and three- ringed PAHs are susceptible to microbial mineralization under ferric iron-reducing, sulfate-reducing, and methanogenic conditions [4,5]. Reproducible evidence of nitrate-reducing PAH mineralization has yet to be reported [5]. Similarly, there is no evidence to date demonstrating that ≥ 4-ringed PAHs can be metabolized anaerobically as a sole carbon source, though co-metabolic PAH degradation is possible [6,7].

Recent efforts have shifted towards profiling the molecular landscape of anaerobic PAH degradation by conducting genomic and proteomic surveys of PAH-degrading cultures. Most of our biochemical understanding of anaerobic PAH biodegradation have been gleaned from the marine isolate NaphS2 and the freshwater enrichment culture N47 [8,9,10,11]. NaphS2 and the dominant organism of N47 are unclassified sulfate-reducing *Desulfobacteraceae* belonging to the *Deltaproteobacteria*, sharing organizational and sequence homology across two gene clusters differentially expressed during naphthalene and 2-methylnaphthalene degradation [12,13,14]. One gene cluster harbors a UbiD-like (de)carboxylase putatively responsible for the carboxylation of naphthalene, while the second gene cluster encodes for the fumarate adding catalytic subunit of naphthyl-2-methylsuccinate synthase (NmsA). Several enzymes predicted to facilitate downstream degradation steps of naphthalene and 2-methylnaphthalene have also been tentatively identified or experimentally verified [10,12,13,14,15,16,17], including the novel de-aromatization enzyme 2-naphthoyl-CoA reductase and the predicted 5, 6-dihydro-2-naphthoyl CoA reductase [18,19,20]. The partial elucidation of these pathways has also helped to identify signature metabolites and functional genes that can serve as diagnostic markers of anaerobic PAH biodegradation in the environment [5,21]. Nevertheless, these model strains only represent a small fraction of the metabolic potential and physiology of organisms likely involved in anaerobic PAH degradation.

Methanogenic PAH degradation has been investigated for over a decade, providing analytical evidence of methyl-substituted naphthalene mineralization and/or microbial community data [22,23,24,25,26,27,28,29,30,31]. However, the identification of catabolic genes, mechanisms, and key microbes involved in methanogenic PAH metabolism has remained largely elusive. A major challenge associated with studying anaerobic PAH degradation is the relatively slow growth rates of PAH degraders, complicating cultivation-dependent biochemical assays [4,5]. This is especially problematic for methanogenic incubations, wherein thermodynamic constraints restrict energy yields for growth and metabolism [5,32]. Instead, cultivation-independent molecular methods such as nucleic acid-based stable isotope probing (SIP) may offer provisional solutions for pinpointing PAH-degrading microorganisms and help to elucidate (novel) anaerobic PAH activation mechanisms. For example, DNA-SIP was recently conducted for an iron-reducing enrichment, revealing that *Thermoanaerobacteraceae* and *Desulfobacterales* phylotypes are involved in 1-methylnaphthalene degradation [33]. However, identifying primary PAH degraders by SIP in complex consortia is difficult as long incubation times generally increase the risk of labeling secondary degraders [34,35]. To our knowledge, only one study has previously used DNA-SIP to monitor PAH degradation under methanogenic conditions, wherein the authors identified three unexpected *Proteobacteria* (*Methylibium*, *Legionella*, *Rhizobiales*) isotopically enriched during methanogenic ^13^C_6_-anthracene degradation [30].

In this study, we aimed to identify key microorganisms and reactions responsible for the initial degradation steps of the PAH naphthalene by a new methanogenic naphthalene-degrading enrichment culture (designated NDC) using a variety of molecular ecology toolbox approaches including DNA-SIP and metagenomic analyses. The culture described in this study originated from a crude oil-degrading enrichment [36,37] found to metabolize methyl-substituted naphthalenes to near stoichiometric amounts of methane [22]. Naphthalene was not initially reported to be susceptible to methanogenic degradation by this culture, but enhanced methane production and naphthalene loss was ultimately verified by gas chromatography more than a year after the original publication date [38]. The results of this present study revealed that members of the *Clostridiaceae*, and possibly *Desulfuromonadales*, are involved in naphthalene degradation by NDC under methanogenic conditions, as these phylotypes became labeled during incubations with isotopically heavy naphthalene and/or harbored genes known to be involved in anaerobic naphthalene metabolism.

## 2. Materials and Methods

### 2.1. Culture Establishment

Preliminary attempts to establish the present methanogenic naphthalene-degrading enrichment culture were first described by Berdugo-Clavijo et al. [22], but new details are presented here. Parent cultures were prepared by adding 3 mL of resuspended pelleted cells from a crude oil-degrading culture [37] to 60 mL of sterile, anoxically prepared bicarbonate-buffered minimal salts medium containing resazurin as a redox indicator and reduced with 0.05% *w*/*v* cysteine sulfide. Triplicate microcosms were amended with 8 mg naphthalene pre-adsorbed in 0.3 g of resin (Amberlite XAD-7 [39]), and incubated at room temperature (~22 °C) under dark and static conditions. Substrate-free controls containing resin only and cell-free sterile controls were also established in triplicate, for a total of nine microcosms. To monitor methanogenic activity, microcosm headspaces were routinely measured for methane production by gas chromatography (GC) [22]. After 140 days of incubation, naphthalene-amended enrichments had only produced equal or lesser amounts of methane relative to substrate-free microcosms (Figure S1).

In order to further verify whether naphthalene could be used as a substrate by this methanogenic culture, the enrichment (10% *v*/*v*) was transferred into 70 mL of fresh medium. Two microcosms were amended with 58.5 mM naphthalene, and dissolved in 1 mL of the inert carrier 2,2,4,4,6,8,8-heptamethylnonane (HMN). HMN, rather than resin, was used in order to enable the measurement of naphthalene loss over time and to assess stoichiometric yields of methane over time. Two unamended microcosms (containing HMN only) and a sterile control were also prepared, for a total of five microcosms. We verified that no naphthalene carry-over had occurred in the unamended controls (see below). Incubation time and temperature were increased to 320 days and 30 °C respectively, to help stimulate methanogenic activity.

Naphthalene loss was measured by injecting 1 µL of the HMN overlay into an Agilent 7890A GC equipped with an HP-5 capillary column (30 m × 320 µm × 0.25 µm; Agilent, Santa Clara, CA, USA) and a flame ionization detector (GC-FID). The oven was held at 50 °C for 2 min, then increased at a rate of 7 °C min to 140 °C, and held at this temperature for 3 min. The injector, operated in split mode (50:1), was held at 275 °C and the detector was held at 300 °C. Calibration curves were prepared from naphthalene standards of known concentrations dissolved in HMN (2–80 mM).

### 2.2. Metabolite Analysis and ^13^C-bicarbonate Tracer Experiments

Subsamples of active NDC enrichments (≤50 mL) were periodically extracted for putative naphthalene metabolites and analyzed by gas chromatography–mass spectrometry (GC-MS) [22]. We also prepared a series of ^13^C-bicarbonate tracer incubations to search for evidence of a carboxylation activation mechanism. To prepare the inoculum, pelleted cells from 75 mL of NDC were washed and resuspended in an equal volume of anoxic medium buffered with 25 mM HEPES solution. Incubations containing 70 mL fresh medium were inoculated with 3 mL of HEPES-buffered inoculum and amended with 19.5 mM naphthalene in HMN and 23 mM of ^13^C-sodium bicarbonate (99%; Cambridge Isotope Laboratories, Inc., Tewksbury, MA, USA) as a co-substrate in a N_2_ headspace. Cultures were incubated at room temperature for over a year before they were extracted and analyzed for putative ^13^C-labeled metabolites by GC-MS.

### 2.3. Microbial Community Analysis and Targeted Functional Gene Analysis

An actively-degrading methanogenic naphthalene enrichment culture was subsampled (4 mL) and extracted for genomic DNA using a commercial kit (FastDNA SPIN Kit for Soils, MP Biomedicals, Solon, OH, USA) according to the manufacturer’s procedure. PCR-based targeted gene surveys assays of the extracted DNA were performed to search for putative anaerobic naphthalene degradation genes, summarized in Table S1. PCR reactions (50 µL) were prepared using the Taq DNA Polymerase kit (Qiagen, Toronto, ON, Canada) and contained 2 µL of template DNA (0.2 ng/µL). 16S rRNA gene sequencing (by 454 pyrotag sequencing) of extracted genomic DNA was carried out using an established two-step PCR method targeting the V6-V8 regions of the 16S rRNA gene [22]. Purified amplicons of expected size were sequenced using a GS FLX Titanium Series Kit XLR70 (Roche Diagnostics Corporation, Indianapolis, IN, USA), and data analysis was conducted in Phoenix 2 as previously described [22]. Pyrosequencing reads are available within NCBI’s Short Read Archive (SRA) under BioProject PRJNA478574.

### 2.4. ^13^C-Naphthalene Stable Isotope Probing

DNA-based SIP was conducted to help pinpoint key naphthalene degraders in NDC. This methodology was selected in particular because it offers higher taxonomic information than protein-SIP, and ^13^C-labeled DNA is less challenging to isolate in sufficient quantities than ^13^C-labeled mRNA [35,40]. Due to limited volumes of available culture and slow growth rates, SIP microcosms were prepared using 10 mL of active, undiluted culture (100% transfer *v*/*v*) and amended (in triplicate) with 4 mg unlabeled [^12^C_10_] or fully labeled [^13^C_10_] naphthalene (99 atom %; Sigma Aldrich, St. Louis, MO, USA) dissolved in 2 mL of HMN. Substrate-unamended (containing HMN only) and sterile control replicates (fresh medium containing ^12^C-naphthalene) were also established, for a total of eight experimental microcosms.

Once a month, for a total of three months, a ^12^C and ^13^C replicate were sacrificed for SIP analysis following the procedures outlined by Neufeld et al. [40]; unamended and sterile replicates were harvested after three months of incubation. DNA was extracted as before and quantified using Qubit fluorometry (Invitrogen, Carlsbad, CA, USA). Up to 227 ng of DNA were retrieved per 10 mL replicate and combined with cesium chloride (density = 1.88 g/mL) and gradient buffer in polyallomer Quick-Seal centrifuge tubes (Beckman Coulter, Brea, CA, USA). Samples were centrifuged at 50,000 rpm (∼300,000 × g_av_) in a VTi 90 rotor (Beckman Coulter, Brea, CA, USA) at 20 °C with vacuum for 65 hours. DNA was retrieved by density fractionation resulting in an average of 12 fractions (425 µL each), where fraction 1 was the heaviest and fraction 12 was the lightest. The density of each fraction was measured with a refractometer (AR200, Reichert, Depew, NY, USA) to confirm proper gradient formation. DNA was precipitated from the CsCl with polyethylene glycol and glycogen, washed with 70% ethanol, and eluted in 30 µL Tris-EDTA buffer. 

Following density gradient fractionation, most DNA concentrations could no longer be determined directly by fluorometry. A preliminary attempt to quantify the distribution of total 16S rRNA genes in SIP fractions was performed using a previously described qPCR assay [41], but most samples failed to amplify above threshold levels (10^2^ copies/mL) even after 40 cycles (data not shown). Instead, we sought to identify SIP fractions containing any detectable amounts of DNA by amplifying the V6-V8 regions of 16S rRNA gene fragments using a PCR assay adapted for low concentrations of DNA [42]. Here, PCR reactions (25 µL) contained 12.5 µL 2× PCR Master Mix (Fermentas, Waltham, MA, USA), 9 µL RNase-free water, 2.5 µL gDNA template, and 0.5 µL each (10 µM) of universal primers 926Fi5 (TCGTCGGCAGCGTCAGATGTGTATAAGAGACAGAAACTYAAKGAATTGACGG) and 1392Ri7 (GTCTCGTGGGCTCGGAGATGTGTATAAGAGACAGACGGGCGGTGTGTRC). PCR assays were performed using a three-step thermoprofile: an initial denaturation at 95 °C for 5 min; 25 cycles of 95 °C (40 s), 55 °C (2 min), 72 °C (1 min); and a final extension at 72 °C for 7 min. Amplification of the target region was confirmed on a 1% agarose gel, purified using the Agencourt AMPure XP magnetic bead system (Beckman Coulter, Brea, CA, USA) according to the manufacturer’s protocol, and quantified as before. The relative abundance of reportable 16S rRNA gene amplicons was then plotted against each fraction’s CsCl buoyant densities. ^13^C ‘light’ fractions were identified based on overlapping densities with either ^12^C-naphthalene replicates or unamended fractions. In contrast, ‘heavy’ fractions exclusively detected in ^13^C-naphthalene enrichments were considered as candidates for having incorporated the isotopic label into their genetic material. 16S rRNA amplicons of the selected light and heavy fractions were pooled into separate volumes and processed for subsequent amplification and Illumina sequencing and analysis as previously described [43]. Trimmed ^13^C-labeled OTU sequences were queried against the NCBI non-redundant nucleotide database using BLASTn to identify homology to anaerobic benzene/PAH degraders and other representative organisms, and used to build bootstrapped maximum likelihood trees (500 replicates) in MEGA7 [44]. ^13^C-labeled OTU sequences have been submitted to NCBI under the accession numbers MH548302–MH548312, and raw reads retrieved from all SIP microcosms are available under BioProject PRJNA478574.

### 2.5. Metagenomic Sequencing, Binning, and Genomic Annotation

Three ^13^C ‘heavy’ fractions were identified after two months of incubation (relative to control replicates) and were further processed for metagenomic sequencing. Briefly, 1 ng of ^13^C-labeled DNA (pooled from all three ‘heavy’ fractions) was used to prepare a metagenomic sequencing library using the Nextera XT DNA Library Prep Kit (Illumina, San Diego, CA, USA). Library quality was assessed using a Bioanalyzer High Sensitivity Chip (Agilent, Santa Clara, CA, USA). The library was then sequenced using MiSeq technology (Illumina), obtaining a total of 12.85 million reads. Raw reads were processed with BBmap (http://jgi.doe.gov/data-and-tools/bbtools/): phiX contaminants, residual adapters and low-quality ends were trimmed and removed with bbduk.sh set to the following parameters: k = 23, ktrim = r, kmin = 11, trimq = 20. Bulk assembly of processed reads was performed using SPAdes v3.9.1 [45]. The resulting scaffolds were analyzed and binned using MetaBAT v0.32.4 under default parameters [46]. Each bin was queried by BLASTN against the NCBI non-redundant database to validate functional identity and to provide taxonomic information. The BLASTN outputs were processed with MEtaGenome ANalyzer (MEGAN v.5.11.3) for taxonomic assignment [47], and matched to most of the dominant phylogenies appearing in the respective 16S rRNA microbial community analysis. Annotation of the complete metagenome and draft genomes was conducted using the Integrated Microbial Genomes & Metagenomics (IMG/M) system of the Joint Genome Institute (JGI) website and submitted to the IMG database under GOLD study Gs0127588, “Stable isotope and metagenomic profiling of a methanogenic naphthalene-degrading enrichment culture”.

## 3. Results and Discussion

### 3.1. Analytical Evidence of Methanogenic Naphthalene Degradation

**Figure 1:**
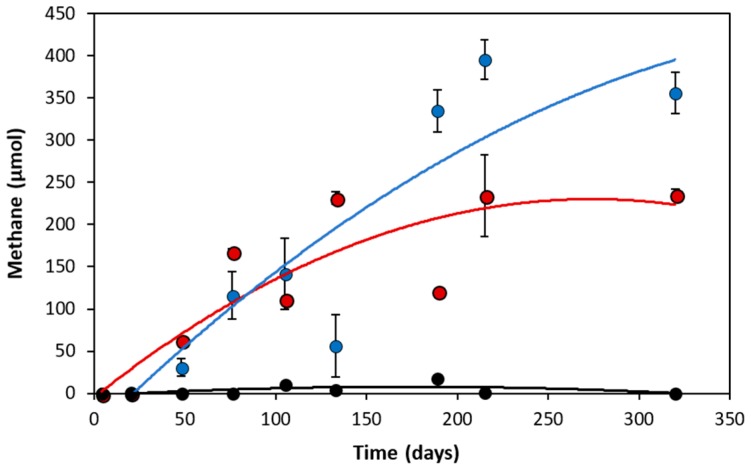
Methane production from transferred NDC incubations amended with naphthalene (blue) relative to substrate-unamended (red) and sterile (black) controls. Bars indicate ± average of duplicate microcosms.

Initial anaerobic PAH degradation investigations conducted by Berdugo-Clavijo et al. [22] suggested that naphthalene could not be metabolized as a methanogenic substrate as similar amounts of methane were produced in substrate-unamended controls compared to naphthalene-amended incubations. However, after transferring the culture into fresh medium and adjusting its incubation conditions, enhanced methane production (up to 162 µmol) was observed in duplicate naphthalene-amended incubations relative to unamended controls; negligible amounts of methane were detected in the naphthalene-amended sterile replicate (Figure 1).

Methane production in the unamended controls (235 µmol) was likely due to metabolism of the cysteine-containing reducing agent. Based on the theoretical stoichiometric conversion of cysteine to methane (4 C_3_H_7_NO_2_S + 6 H_2_O + 4 H^+^ → 4 H_2_S + 4 NH_4_^+^ + 5 CH_4_ +7 CO_2_), the amount of cysteine sulfide added as a reductant may result in the production of up to 222 µmol methane. The balance of carbon could have originated from other trace organics (e.g. dead biomass, trace residual naphthalene) in the growth medium. Accounting for these background methane levels, naphthalene-amended NDC cultures yielded near-stoichiometric amounts of methane (101%) for their reported 20.5 µmol substrate loss (C_10_H_8_ + 8 H_2_O → 6 CH_4_ + 4 HCO_3_^-^ + 4 H^+^), which is comparable to methanogenic methylnaphthalene-degrading enrichment cultures (2-MN and 2-6-dimethylnaphthalene; 2, 6-diMN) maintained by our laboratory [22].

### 3.2. Detection of Putative Naphthalene Metabolites

Based on our GC-MS detection limits (≥10 nM), no metabolites indicative of carboxylation (naphthoic acid), fumarate addition ((methyl)naphthylsuccinic acid), or methylation (methyl naphthalene) were detected during active naphthalene degradation or in ^13^C-bicarbonate tracer experiments. This was supported by the results of our targeted functional gene assays of NDC, wherein no PCR amplification products were obtained using functional primers targeting fumarate addition (*nmsA*) [48] or using an in-house designed primer set targeting aromatic ring carboxylation (*abcA*) [49] (Table S1). We were also unable to detect evidence of the dearomatization gene 2-naphthoyl-CoA reductase using *Ncr* primers [50]. As these PCR-based assays were designed to target highly conserved regions within sulfate-reducing *Deltaproteobacteria* isolates and enrichment cultures, they may be unable to detect a broader diversity of related functional markers in other taxonomic lineages.

**Figure 2:**
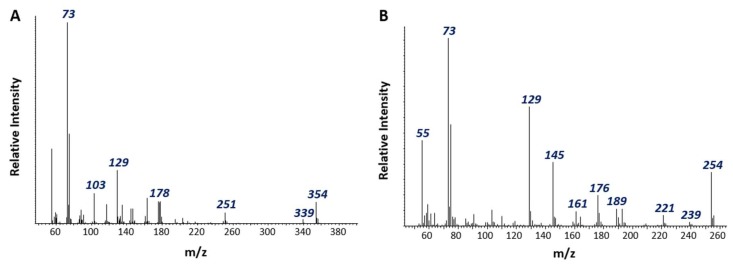
Mass spectra of silylated GC-MS peaks tentatively identified as (**A**) cyclohexane dicarboxylic acid and (**B**) an isomer of deahydro-2-naphthoic acid detected in NDC.

A silylated organic acid tentatively identified as a cyclohexene dicarboxylic acid (M = 354, M − 15 = 339) was detected exclusively in actively degrading enrichment cultures (Figure 2A). Notably, the free acid form of the putative metabolite was 2 mass units less than a cyclohexane dicarboxylic acid (C_11_H_16_O_4_) previously detected during naphthalene degradation by culture N47 [51]. We also detected a peak tentatively identified as decalin carboxylic acid (M = 254, M – 15 = 239) in PAH-amended NDC cultures (Figure 2B). The mass spectrum and GC retention time of this compound differed from those of an authentic standard of decahydro-2-naphthoic acid but could be an isomer. Although decahydro-2-naphthoic acid and its isomeric structures are thought to be dead-end products of anaerobic naphthalene metabolism [5], both putative metabolites suggest that naphthalene degradation by methanogenic consortium NDC undergoes a series of hydrogenation and ring cleavage reactions not unlike those catalyzed by NaphS2 and strain N47.

### 3.3. Pinpointing Putative Naphthalene Degraders Using Pyrosequencing and DNA-SIP

**Figure 3:**
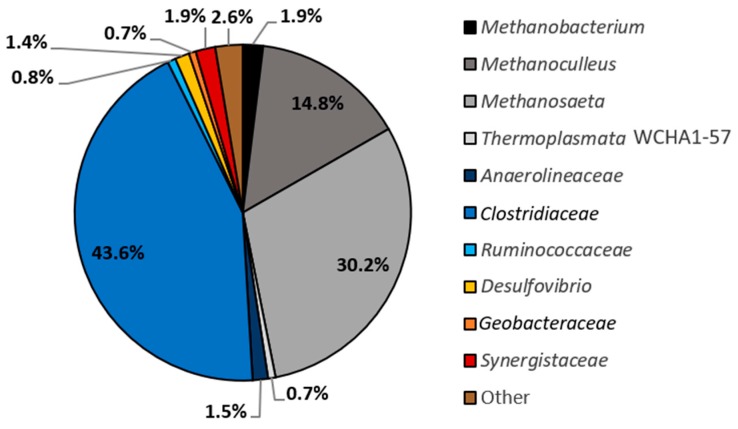
Distribution of bacterial and archaeal 16S rRNA gene pyrotag sequences in an active naphthalene-degrading replicate of NDC. Values are shown as the percentage of sequences per taxon based on 2034 bacterial sequences and 1881 archaeal sequences. OTUs included in ‘Other’ make up less than 0.4% of prokaryotic sequences each and include *Spirochaeta* (0.26%), *Proteiniphilum* (0.13%), and *Candidatus Methanoregula* (0.36%).

Pyrotag sequencing of 16S rRNA genes amplified from NDC revealed the predominance of bacterial OTUs belonging to *Clostridiaceae* (43.6% of reads) (Figure 3). This was not surprising, as members of this family were also heavily enriched in 2-MN and 2, 6-diMN-degrading methanogenic cultures (24.0% and 32.2% of reads, respectively) [22]. Methanogenic archaeal sequences belonging to *Methanosaeta* (30.2% of reads) and *Methanoculleus* (14.8% of reads) were also present in high abundance. All other OTUs recovered from NDC were only minor constituents (<2% read abundance) and collectively comprised less than 12% of reads.

Stable isotope probing with ^13^C-naphthalene and ^12^C-naphthalene as a control was performed to help pinpoint key organisms in NDC that actively participate in methanogenic naphthalene degradation. Triplicate microcosms were sacrificed and harvested for DNA in one month intervals to search for the earliest possible labeling of naphthalene degraders and to minimize risk of secondary cross-feeding. Up to 227 ng of gDNA was extracted from each microcosm, or ~80% less nucleic acid material than was successfully fractionated by Abu Laban et al. [52] during DNA-SIP surveys of a methanogenic toluene-degrading culture originating from an oil sands tailings pond. Note that this is still considerably less than the 5–10 µg of genetic material recommended for density gradient fractionation to ensure proper banding efficiency of labeled and unlabeled nucleic acid species [40]. While low DNA concentrations had no apparent effect on density gradient formation or fractionation, DNA could not be accurately quantified using conventional qPCR techniques after being separated across a dozen fractions (<10^2^ 16S rRNA gene copies/mL). We therefore chose to assess fractions for the presence of amplifiable 16S rRNA gene fragments using PCR as a semi-qualitative substitute for qPCR analysis.

**Figure 4:**
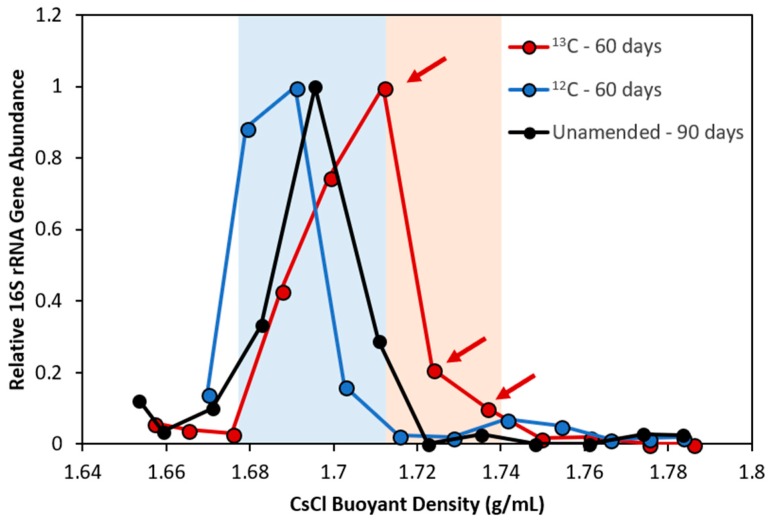
Relative distribution of total 16S rRNA gene fragments in NDC SIP fractions containing ^13^C- and ^12^C-DNA extracted after 60 days of incubation. The red shaded area indicates fractions considered to be enriched in ^13^C-DNA compared to background ^12^C-DNA microorganisms (shaded area in blue) and were predicted to harbor naphthalene degraders. Fractions chosen and pooled for metagenomic sequencing analysis are indicated with arrows.

After two months of incubation, three ‘heavy’ fractions were recovered (1.712–1.736 g/mL) from gDNA extracted from a [^13^C_10_] naphthalene microcosm, harboring OTUs belonging primarily to *Desulfuromonadales* (68.5% of reads) and *Clostridiaceae* (14.5% of reads) (Figure 4, Table 1). A similar microbial community composition was reported in the unlabeled [^12^C_10_] naphthalene control (Figure 4), supporting that these organisms were indeed the most abundant (and presumably most active) members in NDC at this time point. Recovered [^13^C_10_] naphthalene ‘light’ fractions (1.681–1.704 g/mL) contained a 9.52% lower abundance of *Clostridiaceae* than in corresponding ‘heavy’ fractions, showing that this taxon became enriched with the isotopic material, likely due to its involvement in naphthalene metabolism. Though the recovered [^13^C_10_] naphthalene ‘light’ fractions showed a 12.5% greater abundance of *Desulfuromonadales* compared to the ‘heavy’ fractions but its high relative abundance in NDC in general suggests it may also play a role in naphthalene metabolism.

Three minor constituents of NDC (*Methanoculleus*, 0.72%; *Desulfovibrio*, 0.43%; *Clostridium*, 0.27%) may have also been labeled with heavy isotope, though their low abundances make this observation speculative at best (Table 1). A problem arises, particularly for tracking nucleic acids, when substrate incorporation and incubation time are insufficient, generating poorly labeled biomarker molecules that are near-indistinguishable from a background of (relatively abundant) unlabeled molecules [34]. That said, no new ^13^C-labeling was observed after three months of SIP incubation. Determination of isotopic labeling via buoyant densities has a detection limit for ^13^C stable isotope incorporation of ~20 atom% [35], so we may have been very close to this threshold.

**Figure 5:**
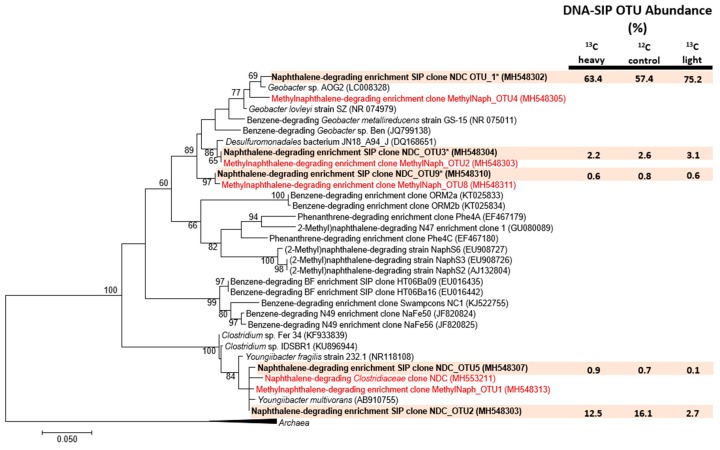
Maximum likelihood consensus tree showing the affiliation of putative ^13^C-labeled sequencing OTUs comprising at least 0.5% abundance (bold, shaded) to methanogenic (methyl)naphthalene pyrosequencing clones (red), known anaerobic benzene/PAH degraders and other published reference strains. Evolutionary analyses of aligned nucleotide sequences were conducted in MEGA7; the consensus tree was constructed using the Tamura–Nei model at all nucleotide positions (528 in total) and performing 500 bootstrap replicates (values below 60% are not shown). Archaeal 16S rRNA gene sequences retrieved from NCBI were used to root the tree (collapsed). Asterisks indicate sequences where more than one clone was obtained and a single representative sequence (accession number) was selected for tree construction.

It was unclear why *Desulfuromonadales* became enriched in both [^13^C_10_] and [^12^C_10_] naphthalene-amended microcosms (Table 1), having only comprised a small fraction (0.7%) of previous pyrosequencing reads (shown as *Geobacteraceae*, Figure 3). Active members of the *Desulfuromonadales* are not typically detected in methanogenic enrichment cultures. Though this family has occasionally been identified in methane-producing coal-bed materials [53,54], their community role(s) have yet to be determined. Marozava et al. [33] identified a member of this family (*Desulfobulbaceae*) to incorporate a ^13^C-carbon label from SIP of an iron-reducing, 1-methylnaphthalene-degrading consortium, predicted to catalyze Fe (III) reduction rather than degrade PAHs. The dominant *Desulfuromonadales* OTU in NDC (NDC OTU_1) appears to more phylogenetically related to acetate-oxidizing *Geobacter lovleyi* strain SZ and *Geobacter* sp. AOG2 (87.0% sequence identity to each) than to known hydrocarbon-degrading isolates (84.6–84.8% sequence identity) (Figure 5). Overall, despite its high relative abundance in NDC, the possible role of *Desulfuromonadales* in naphthalene degradation is ambiguous given our pyrosequencing and DNA-SIP results (and metagenome analysis, see below).

Recovered ^13^C-labeled SIP and pyrosequencing OTUs belonging to *Clostridiaceae* shared 86–99% nucleotide sequence identity to cultured representatives of the *Youngiibacter* (formerly known as *Acetivibrio*), a recently described genus capable of fermenting a broad range of carbohydrates and aromatic acids [55,56,57] (Figure 5). The OTUs also share 84–99% sequence identity to the dominant *Firmicutes* detected in 2-MN and 2, 6-diMN [22]. This clade appears to be phylogenetically distinct from *Clostridium* and does not cluster with any other known or predicted PAH degraders, including the three anthracene-degrading SIP clones recovered by Zhang et al. [30] and the *Thermoanerobacteraceae* identified by Marozova et al. [33].

### 3.4. Metagenomic Evidence Predicts *Clostridiaceae* to Be a Key Naphthalene Degrader in NDC

Whole metagenomic shotgun sequencing and assembly of pooled ^13^C-labeled fractions was performed to generate a primary culture metagenome (IMG Genome ID 3300013290) and 10 supporting draft genomes (Table 2). The final metagenome consisted of an estimated 51212 scaffolds encoding 103278 protein-coding genes, of which roughly one-third were not orthologous to known proteins. A single *Clostridiaceae* sp. bin was assembled from the metagenomic sequences, predicted to be NDC_OTU2 (Figure 5), and shared >87% protein sequence similarity to *Youngiibacter fragilis* across 54% of its predicted 5424 protein-encoding genes (Table S2). *Y. fragilis* is a strictly anaerobic member of the *Clostridiaceae* family isolated from a natural gas-producing coal bed system [55]. Previous genomic sequencing by Wawrik et al. [56] revealed no evidence of known anaerobic hydrocarbon activation pathways in the pure culture. However, the remaining half of the genomic material in our *Clostridiaceae* bin appears to be weakly similar or unrelated to the type strain.

When comparing annotated protein-encoding genes to connected hydrocarbon metabolism KEGG routes, most draft genomes yielded only single hits and were not considered to be significant. We were also unable to detect any evidence of sequences orthologous to previously proposed anaerobic naphthalene carboxylation genes (*abc*, *ubiX*, *ubiD*), naphthoyl-2-coenzyme A reductase, or fumarate addition mechanisms. Thus, we further sought evidence of downstream anaerobic naphthalene degradation pathways using a curated database of all publicly available predicted anaerobic benzene/PAH degradation genes in NBCI and IMG (as of March 2017). A total of 804 genes of interest (with a minimum e-value cutoff = 10^−5^) were detected across 453 scaffolds in the prepared draft genomes, of which 85% contained less than three naphthalene degradation gene orthologues and were thus removed from further analysis. The remaining 73 candidate scaffolds were manually assessed for functional and structural consistency to predicted/known PAH-degrading genes in strains NaphS2, N47, and in other reference genomes. Most scaffolds were found to encode for predicted Fe-containing hydrogenases (including in the *Desulfuromonadales* bins) and have yet to be investigated further, as their broad functional diversity makes it difficult to discern an exclusive role in anaerobic naphthalene degradation.

**Figure 6:**
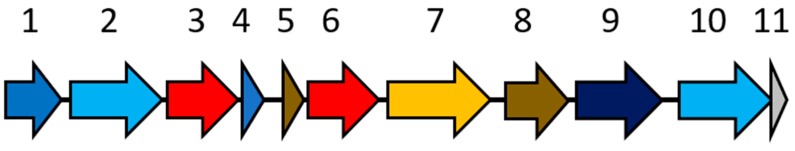
Organization of putative methanogenic naphthalene degradation gene cluster of binned *Clostridiaceae* scaffold 1013. Protein functions were predicted based on sequence orthology to NaphS2 loci and BLASTP searches. Protein annotation: **1.** enoyl CoA hydratase; **2.** Class III CoA transferase; **3.** FAD-dependent arylcarboxyl-CoA reductase; **4.** enoyl-CoA hydratase/isomerase/hydrolase; **5.** 3-hydroxybutyryl-CoA dehydrogenase; **6.** FAD-dependent arylcarboxyl-CoA reductase; **7.** H+/gluconate symporter; **8.** 3-hydroxybutyryl-CoA dehydrogenase; **9.** acetyl-CoA C-acetyltransferase; **10.** Class III CoA transferase; **11.** hypothetical protein.

In the *Clostridiaceae* bin, we identified a gene cluster of key interest (Scaffold 1013) wherein 9 of the 12 predicted protein-encoding genes shared 44–65% protein sequence similarity to *Deltaproteobacterium* strain NaphS2 (Figure 6). The enzymes encoded in Scaffold 1013 are also shown in Table 3 together with their predicted putative function according to sequence homologs in NaphS2 and other organisms using BLASTP. We manually confirmed that Scaffold 1013 contained no orthologous regions to the genome of *Y. fragilis*.

Two genes predicted to encode CoA-transferase Family III proteins were identified in Scaffold 1013 shared 44–61% similarity across a handful of NaphS2 loci and may serve to catalyze CoA-thioesterification in a reaction that is similar but not identical to naphthoyl-CoA ligase (Figure 6, Table 3). Family III CoA-transferases occur in prokaryotes and eukaryotes, and catalyze reversible, ATP-independent CoA-transfer reactions in a highly substrate- and stereo-specific manner [58]. These enzymes operate via a ternary complex mechanism, allowing for recycling of the CoA donor within the enzyme complex and increased energy conservation during substrate catabolism. Scaffold 1013 also encodes for a predicted acetyl-CoA C-acetyltransferase and a predicted H^+^/gluconate symporter (GntP) that could help to accumulate the CoA derivative compounds within the cell [5,58]. Several anaerobic catabolic pathways rely on Family III CoA-transferases to recycle CoA intermediates [55], including succinyl-CoA during anaerobic toluene degradation (succinyl CoA:(*R*)-benzylsuccinate CoA-transferase) [59,60], and 2-naphthoyl-CoA during 2-methylnaphthalene metabolism by NaphS2 and strain N47 (succinyl-CoA: naphthyl-2-methyl-succinate CoA-transferase) [12,61]. Though fumarate addition does not appear to be the mechanism of naphthalene activation by NDC, a similar unknown intermediate may be being used instead. Notably, if the *Clostridiaceae* phylotype is indeed catalyzing the addition of CoA to an unknown naphthalene activation product, then it is plausible that the organism is also chiefly responsible for initiating methanogenic attack of the PAH in NDC.

Scaffold 1013 harbored two predicted flavin-containing enzymes with 48–60% similarity to two NaphS2 orthologs annotated as 2-nitropropane dioxygenases [12] (Figure 6, Table 3). As NDC was maintained under strict anoxic conditions and 2-nitropropane is not a proposed intermediate of anaerobic naphthalene degradation, these annotations are more than likely erroneous. We hypothesize that these protein sequences may instead encode for dearomatizing arylcarboxyl-CoA reductases similar to those of 2-naphthoyl-CoA reductase and 5, 6-dihydro-2-naphthoyl CoA reductase in NaphS2 and N47. However, because the protein-encoding genes are not sequential to one another in Scaffold 1013 (Figure 6) as they are in strain N47 (N47_G38220 and N47_G38210, respectively) and in NaphS2 (NPH_5475 and NPH_5476, respectively), the pathway for reduction of the unknown arylcarboxyl-CoA structure appears to be unique. Enzyme assays conducted by Estelmann et al. [20] showed that predicted 5, 6-dihydro-2-naphthoyl CoA reductases contained flavin adenine dinucleotide (FAD) and flavin mononucleotide (FMN), and that complete reduction of both flavin cofactors (and a [4Fe-4S] cluster) was required to catalyze highly negative (E°′= −493 mV) two-electron reduction of 2-naphthoyl-CoA to 5,6-dihydro-2-naphthoyl-CoA. Therefore, a similar reaction may be required to reduce naphthalene under methanogenic conditions.

Four predicted ß-oxidation-like degradation genes detected in Scaffold 1013 were orthologous to sequences encoded within the proposed tetralin (*thn*) operon in NaphS2 (Figure 6; Table 3). Enzymes encoded by this operon are predicted to catalyze tetrahydronaphthoyl-CoA ring cleavage in NaphS2 and N47 [12,13]. Meckenstock et al. [5] proposed that enoyl-CoA hydratases/isomerases/hydrolases (ThnKLM) and acyl-CoA dehydrogenases (ThnO) facilitated ß-oxidation-like reactions leading towards ring opening, while dehydrogenation reactions following ring cleavage were catalyzed by predicted 3-hydroxyacyl-CoA dehydrogenases (ThnK and ThnY). Given that we also detected tentative metabolite evidence of dearomatization and ring cleavage reactions (Figure 2), it is plausible that *Clostridiaceae* members of NDC may coordinate at least some similar naphthalene degradation reactions under methanogenic conditions. However, another possible function of these enzymes is to catalyze ß-oxidation-like degradation steps of the unknown arylcarboxyl-CoA structure, similar to benzyl- and naphthyl-2-methyl-succinyl-CoA [12,13,61,62]. This would result in the formation of an intermediate aryl-acid CoA-ester similar to 2-napthoyl-CoA that could then be further degraded by *Clostridiaceae*.

## 4. Conclusions

PAH are important components that can persist in anoxic fuel-contaminated environments, thus understanding their natural fate processes, such as their microbial degradation, can aid in environmental clean-up efforts. The results of this present study add to the small body of knowledge describing methanogenic PAH biodegradation. Through the use of 16S rRNA amplicon sequencing, metabolite analysis, DNA-SIP using ^13^C-naphthalene, and assembly-based metagenomics of heavy fractions, our data suggest that a member of the *Clostridiaceae* is a key player in naphthalene metabolism in the culture. Enrichment of a *Desulfuromonadales* phylotype in NDC suggest a possible role for this organism as well, though this remains ambiguous as our metagenomic data did not reveal naphthalene-specific genes in the bins associated with the organism. While metabolite and genomic approaches did not allow us to identify an initial postulated mode of attack of naphthalene (e.g., via carboxylation), we did identify metabolites (tentatively) and genes predicted to encode for enzymes facilitating metabolic processes for downstream catabolic reactions including naphthalene carboxylic acid CoA-thioesterification and ring reduction steps only partially similar to NaphS2 and strain N47. These data suggest that more than one transformation pathway may be used to drive anoxic naphthalene degradation, demanding greater in-depth genomic investigations of diverse anaerobic PAH degrading taxa. Ongoing scale-up efforts of NDC and other naphthalene-degrading methanogenic enrichments that yield increased cell material will allow for additional studies to determine whether initial steps are also similar or different.

## Figures and Tables

**Table 1:** Distribution of the most abundant classified taxa (%) across SIP fractions collected after 60 days of incubation, as determined by 16S rRNA gene sequencing.

Taxon	^13^C Heavy	^12^C Control	^13^C Light
***Archaea***			
*Methanosaeta*	1.49	5.18	4.83
*Methanobacterium*	0.14	0.64	2.19
*Methanoculleus*	0.72	0.17	0.04
*Thermoplasmata* WCHA-57	0.00	0.04	0.35
***Bacteroidetes***			
*Proteiniphilum*	0.07	0.53	0.30
*Rikenellaceae* vadinBC27	0.00	0.17	0.70
***Chloroflexi***			
*Anaerolineaceae*	0.14	1.48	0.73
***Firmicutes***			
*Clostridiaceae*	14.5	17.8	4.98
*Sedimentibacter*	0.07	1.59	0.47
*Clostridium*	0.27	0.07	0.03
*Peptococcaceae*	0.16	0.13	0.01
***Proteobacteria***			
*Desulfuromonadales*	68.5	61.6	81.0
*Geobacter*	1.88	1.70	2.06
*Desulfuromonas*	0.45	1.68	0.36
*Desulfobulbus*	0.32	0.42	0.54
*Desulfovibrio*	0.43	0.44	0.11
*Deltaproteobacteria*	0.41	0.15	0.21

^12^C Control: indicates cultures amended with unlabeled naphthalene that were incubated alongside those amended with isotopically labeled naphthalene.

**Table 2:** Genome sequence similarity of prepared draft genomes to their closest matching cultured representative or enriched strain. Pairwise average nucleotide identity (ANI) calculations were used to assess the bidirectional best hits (BBH) of genes having 70% or more identity to reference strains and at least 70% coverage of the shorter gene. Alignment fraction (AF) calculations were prepared to determine the % coverage of the draft genomes to reference strains.

Genome1 IMG ID	Genome1 Name	Genome Size, bp (Assembled)	Gene Count (Assembled)	Genome2 IMG ID	Genome2 Name	ANI 1→2	ANI 2→1	AF 1→2	AF 2→1	Total BBH
2724679725	*Anaerolinea* sp. Bin 1	2333788	2249	649633005	*Anaerolinea thermophila* UNI-1	67.6	67.6	0.08	0.05	221
2724679726	*Anaerolinea* sp. Bin 2	2271625	2153	649633005	*Anaerolinea thermophila* UNI-1	67.7	67.7	0.13	0.08	318
2724679696	Unclassified *Clostridiaceae* sp. Bin 1	5438132	5424	2582580929	*Youngiibacter fragilis*	87.5	87.5	0.54	0.75	2904
2724679721	*Desulfovibrio* sp. Bin 1	3088242	2959	2561511137	*Desulfovibrio alcoholivorans* DSM 5433	72.6	72.7	0.40	0.25	1337
2724679697	Unclassified *Desulfuromonadales* sp. Bin 1	4390079	4104	640427115	*Geobacter uraniireducens* Rf4	72.4	72.4	0.29	0.25	1225
2724679723	Unclassified *Desulfuromonadales* sp. Bin 2	3876684	3546	640427115	*Geobacter uraniireducens* Rf4	72.2	72.2	0.35	0.26	1230
2724679724	Unclassified *Desulfuromonadales* sp. Bin 3	2526538	2519	642555130	*Geobacter lovleyi* SZ	77.0	77.0	0.69	0.43	1737
2724679722	*Methanosaeta* sp. Bin 1	2683322	2798	650716054	*Methanosaeta concilii* GP-6	96.6	96.6	0.81	0.76	2209
2724679727	*Sphaerochaeta* sp. Bin 1	1750954	1956	650377973	*Sphaerochaeta globosa* Buddy	80.5	80.5	0.70	0.37	1321
2724679720	Unclassified *Rhodospirillales* Bin 1	3473168	3651	2651870079	Unclassified *Rhodospirillaceae* Bin 35	69.5	69.5	0.15	0.14	546

**Table 3:** Overview of binned *Clostridiaceae* Scaffold 1013. Percent sequence identity/similarity of protein-encoding encoding genes are compared to orthologous loci detected in the NaphS2 genome and within NCBI protein sequence database.

Gene ID	IMG Predicted Protein Annotation	Length (aa)	NaphS2 Ortholog	Locus	% Identity (% Similarity)	Best BLASTP Ortholog	Accession No.	% Identity (% Similarity)
2727804033	Hypothetical protein	60	N/A	-	-	hypothetical protein [*Clostridiales* bacterium VE202-03]	WP_024723634	43 (71)
2727804034	Crotonobetainyl-CoA:carnitine CoA-transferase CaiB	412	CoA-transferase family III protein	NPH_4605 NPH_6726 NPH_5274	31 (50) 24 (46) 27 (44)	Formyl-coenzyme A transferase [uncultured *Clostridium* sp.]	SCJ80195	55 (74)
2727804035	acetyl-CoA C-acetyltransferase	387	Acetyl-CoA C-acyltransferase	NPH_3581 NPH_6994 NPH_6993 NPH_5273	43 (61) 43 (62) 38 (62) 38 (55)	MULTISPECIES: acetyl-CoA acetyltransferase [*Geobacillus*]	WP_074043744	51 (69)
2727804036	3-hydroxybutyryl-CoA dehydrogenase	288	3-hydroxybutyryl-CoA dehydrogenase	NPH_5812 NPH_5906 NPH_5896 NPH_7219	38 (58) 40 (56) 36 (53) 34 (53)	3-hydroxyacyl-CoA dehydrogenase [*Clostridiales* bacterium PH28_bin88]	KKM10089	53 (71)
2727804037	H+/gluconate symporter	444	N/A	-	-	MULTISPECIES: hypothetical protein [*Clostridiales*]	WP_007862308	43 (65)
2727804038	enoyl-[acyl-carrier protein] reductase II	315	2-nitropropane dioxygenase	NPH_5508 NPH_6957	35 (52) 33 (53)	2-nitropropane dioxygenase [*Anaerosporomusa subterranea*]	WP_066237624	52 (70)
2727804039	Enoyl-CoA hydratase/isomerase	85	3-hydroxybutyryl-CoA dehydratase	NPH_5887 NPH_5907	47 (61) 40 (56)	MULTISPECIES: 3-hydroxybutyryl-CoA dehydratase [*Clostridiales*]	WP_007862300	52 (74)
2727804040	Enoyl-CoA hydratase/isomerase	78	3-hydroxybutyryl-CoA dehydratase	NPH_6695 NPH_5897 NPH_5898	39 (62) 38 (63) 45 (65)	enoyl-CoA hydratase [*Desulfotomaculum thermosubterraneum*]	WP_072869855	55 (77)
2727804041	2-nitropropane dioxygenase precursor	321	2-nitropropane dioxygenase	NPH_6957 NPH_5508	38 (60) 31 (48)	nitronate monooxygenase [*Clostridium citroniae*]	WP_083424018	71 (84)
2727804042	Crotonobetainyl-CoA:carnitine CoA-transferase CaiB	409	CoA-transferase family III protein	NPH_4605 NPH_1868	34 (52) 26 (44)	MULTISPECIES: CoA transferase [*Clostridiales*]	WP_007862305	48 (68)
2727804043	2-(1,2-epoxy-1,2-dihydrophenyl)acetyl-CoA isomerase	262	putative enoyl-CoA hydratase	NPH_0885 NPH_5898 NPH_5897	31 (50) 32 (54) 32 (52)	enoyl-CoA hydratase [*Enterococcus silesiacus*]	WP_071879306	47 (71)
2727804044	Hypothetical protein	25	N/A	-	-	ATP-dependent endonuclease [*Bacillus cereus*]	OPA11549	72 (77)

## Supplementary Materials

The following are available online at http://www.mdpi.com/2076-2607/6/3/65/s1, Figure S1: Methane production from triplicate parent NDC microcosms amended with naphthalene (blue) relative to an unamended control (red), Table S1: Primer sets screened for the targeted detection of anaerobic functional genes in a methanogenic naphthalene-degrading enrichment culture, Table S2: Summary of draft genomes assembled from pooled ^13^C-labeled NDC SIP fractions.
