# Supplementary material for: Stable Isotope and Metagenomic Profiling of a Methanogenic Naphthalene-Degrading Enrichment Culture
Source: Microorganisms. 2018 Jul 10;6(3):65. doi: 10.3390/microorganisms6030065 (PMC6164631; doi:10.3390/microorganisms6030065)
Supplement: Supplementary file 1 [file microorganisms-06-00065-s001.pdf]

Stable Isotope and Metagenomic Profiling of a Methanogenic Naphthalene-Degrading Enrichment Culture

Courtney R. A. Toth, Carolina Berdugo-Clavijo, Corynne M. O'Farrell, Gareth M. Jones, Andriy Sheremet, Peter F. Dunfield, and Lisa M. Gieg

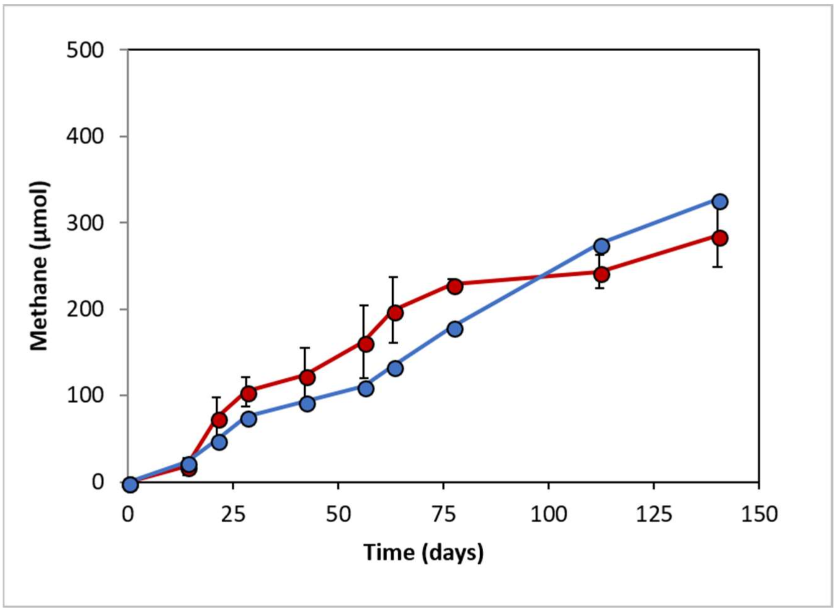

Figure S1. Methane production from triplicate parent NDC microcosms amended with naphthalene (blue) relative to an unamended control (red). Adapted from Berdugo Clavijo et al., 2012.

Table S1. Primer sets screened for the targeted detection of anaerobic functional genes in a methanogenic naphthalene-degrading enrichment culture.

Primer name		Target gene	Target lineage	Primer sequence (5' – 3')	Expected amplicon bp	Amplification detected	References
FAE-B	7768f<br>8543r	*bssA*	Clostridial *bssA*, *bssA* s.l., *nmsA*	CAAYGATTTAACCRACGCCAT<br>TCGTCRTTGCCCCAYTTNGG	775	-	(von Netzer et al., 2013)
FAE-N	7363f<br>7374f<br>8543r	*nmsA*	*nmsA* s. str.	TCGCCGAGAATTTCGAYTTG<br>TTCGAYTTGACGGACAGCGT<br>TCGTCRTTGCCCCAYTTNGG	1180<br>1169	-	(von Netzer et al., 2013)
Ncr2$_{for}$<br>Ncr2$_{rev}$		*Ncr*	*Deltaproteobacteria* (NaphS2, N57)	TGGACAAAYAAAMGYACVGAT<br>GATTCCGGCTTTTTTCCAAVT	320	-	(Morris et al., 2014)
benCarb$_{for}$<br>benCarb$_{rev}$		*abcA*		GTGGTCTTCGCACCGTTAAT<br>TCTCCGGTAACTGGGTGTTC	495	-	(Fowler, 2014)

Table S2. Summary of draft genomes assembled from pooled ^{13}C-labelled NDC SIP fractions.

Draft (meta)genome	IMG Genome ID	Genome size (bp)	Gene count	Protein coding genes with COGs (%)	Scaffolds containing ≥1 PAH-degrading gene orthologs	Scaffolds containing ≥3 PAH-degrading gene orthologs
^{13}C-enriched DNA metagenome of NDC	3300013290	68705674	104254	64.54	804	73
Clostridium sp. Bin 1	2724679696	5438132	5424	68.31	260	28
Geobacter sp. Bin 1	2724679697	4390079	4104	56.80	90	9
Geobacter sp. Bin 2	2724679723	3876684	3546	62.15	79	9
Geobacter sp. Bin 3	2724679724	2526538	2519	65.58	58	7
Unclassified *Rhodospirillales* Bin 1	2724679720	3473168	3651	60.81	88	2
Desulfovibrio sp. Bin 1	2724679721	3088242	2959	65.12	71	7
Anaerolinea sp. Bin 1	2724679725	2333788	2249	56.87	52	4
Anaerolinea sp. Bin 2	2724679726	2271625	2153	57.50	34	4
Sphaerochaeta sp. Bin 1	2724679727	1750954	1956	51.74	41	0
Methanosaeta sp. Bin 1	2724679722	2683322	2798	58.26	31	3

References

1. Berdugo-Clavijo, C.; Dong, X.; Soh, J.; Sensen, C.W.; Gieg, L.M. Methanogenic biodegradation of two-ringed polycyclic aromatic hydrocarbons. *FEMS Microbiol. Ecol.* **2012**, *81*, 124–133.
2. Fowler, S.J. Syntrophic hydrocarbon metabolism under methanogenic conditions. PhD Thesis. University of Calgary, Calgary, Alberta, 2014.
3. Morris, B.E.; Gissibl, A.; Kummel, S.; Richnow, H.H.; Boll, M. A pcr-based assay for the detection of anaerobic naphthalene degradation. *FEMS Microbiol. Lett.* **2014**, *354*, 55–59.
4. von Netzer, F.; Pilloni, G.; Kleindienst, S.; Kruger, M.; Knittel, K.; Grundger, F.; Lueders, T. Enhanced gene detection assays for fumarate-adding enzymes allow uncovering of anaerobic hydrocarbon degraders in terrestrial and marine systems. *Appl. Environ. Microbiol.* **2013**, *79*, 543–552.
